# Moara: a Java library for extracting and normalizing gene and protein mentions

**DOI:** 10.1186/1471-2105-11-157

**Published:** 2010-03-26

**Authors:** Mariana L Neves, José-María Carazo, Alberto Pascual-Montano

**Affiliations:** 1 BioComputing Unit, National Center of Biotechnology (CNB-CSIC), Madrid, Spain; 2 Institute of Molecular Medicine Principe de Asturias (IMMPA-CSIC), Madrid, Spain

## Abstract

**Background:**

Gene/protein recognition and normalization are important preliminary steps for many biological text mining tasks, such as information retrieval, protein-protein interactions, and extraction of semantic information, among others. Despite dedication to these problems and effective solutions being reported, easily integrated tools to perform these tasks are not readily available.

**Results:**

This study proposes a versatile and trainable Java library that implements gene/protein tagger and normalization steps based on machine learning approaches. The system has been trained for several model organisms and corpora but can be expanded to support new organisms and documents.

**Conclusions:**

Moara is a flexible, trainable and open-source system that is not specifically orientated to any organism and therefore does not requires specific tuning in the algorithms or dictionaries utilized. Moara can be used as a stand-alone application or can be incorporated in the workflow of a more general text mining system.

## Background

Some of the most important steps in the analysis of scientific literature are related to the extraction and normalization of genes and proteins in the text and their association with the particular entry in a corresponding biological database. These are known as gene/protein recognition and normalization tasks, respectively, and are common preceding steps to complex text mining tasks.

The main difficulties relating to gene/protein recognition and normalization tasks are the large number of existing gene and protein entities and a lack of rules concerning nomenclature, or the resistance of the scientific community to its use [1]. Some entities coincide with common English words (e.g., "deafness"), which complicates their detection in free-form text. In addition, nomenclature can appear as long descriptive names (e.g., "tumor necrosis factor") or as acronyms (e.g., "TNF"), making normalization difficult. Furthermore, some existing biological entities are known by more than one name and some newly-discovered entities have been assigned a name that is already in use for an existing gene or protein.

For gene normalization, different organisms might require different strategies [2] or specific curated dictionaries [3,4], depending on the complexity of their nomenclature and the degree of ambiguity in the assigned synonyms. This is a problem because a name may or may not refer to distinct entities of the same organism. Gene/protein extraction and normalization tasks are extremely important, and have received much attention from the scientific community. BioCreative evaluation [5-7] is one example of a community-wide effort to evaluate text mining systems applied to the field of biology.

Many solutions have been proposed for gene/protein recognition [5] and normalization [6,7] tasks. There are freely available taggers, but a mix of them is desirable in order to extract the maximum mentions of an entity from a text. For gene/protein extraction, Banner [8], ABNER [9] and GENIA [10] produce good results based on Conditional Random Fields [11], as well as the U-Compare framework [12]. Web available systems including GNAT [13] and Whatizit [14] are available for normalization tasks. Despite research into the development of gene/protein recognition and normalization methodologies, the search continues for reliable systems and dictionaries of synonyms that can be easily integrated into more general text mining systems.

Moara, which comes as a freely available Java library, could be an alternative to these systems. Gene/protein recognition is carried out by a Case-based reasoning approach (CBR-Tagger), and a machine learning methodology that uses an organism-independent strategy is proposed (ML-Normalization) for normalization steps. The normalization procedure is currently available for *Saccharomyces cerevisiae *(yeast), *Mus musculus *(mouse), *Drosophila melanogaster *(fly) and *Homo sapiens *(human), but can be trained with new organisms. The results presented in the supplementary material demonstrate the suitability of these strategies for gene/protein tagging and normalization tasks.

## Implementation

The Moara project is a Java library oriented to gene/protein recognition and normalization tasks, carried out by CBR-Tagger and ML-Normalization, respectively. The system makes use of some MySQL databases and three external libraries: the Weka machine learning tool [15], SecondString http://secondstring.sourceforge.net/ library for string distance metrics, and ABNER [9] as an additional tagger for the extraction of mentions.

MySQL databases store data that have been learned by the system during training phases and external data that are necessary for some of the functionalities of the system. The four databases in Moara are listed below:

• **moara**: contains general and biological data that are of use for the functionalities in the project. This database holds the data related to stopwords http://moara.dacya.ucm.es/download.html, Biothesaurus biomedical terms http://pir.georgetown.edu/pirwww/iprolink/biothesaurus.shtml and a list of all organisms present in Entrez Gene Taxonomy http://www.ncbi.nlm.nih.gov/Taxonomy/, and is essential for all functionalities of the Moara project.

• **moara_mention**: contains data (cases) that are learned during the training step of CBR-Tagger; it is used for extracting gene/protein mentions from texts.

• **moara_gene**: contains data related to the genome, and a dictionary of synonyms of the organisms under consideration. The current version supports yeast, mouse, fly and human. This data are used for both the matching procedure and the disambiguation strategy of the gene/protein normalization task.

• **moara_normalization**: contains data related to the transformations that have been applied to the gene/protein synonyms in order to compose the features that take part in the machine learning matching procedure of the normalization task.

This section describes the methodology that was used in the development of both systems, as well as the details of the available functionalities in version 1.0.5 of the Moara Project. For the sake of simplicity only a straightforward example will be shown. The documentation page http://moara.dacya.ucm.es/documentation.html supplies complete code examples.

### Example of use

**Figure 1:**
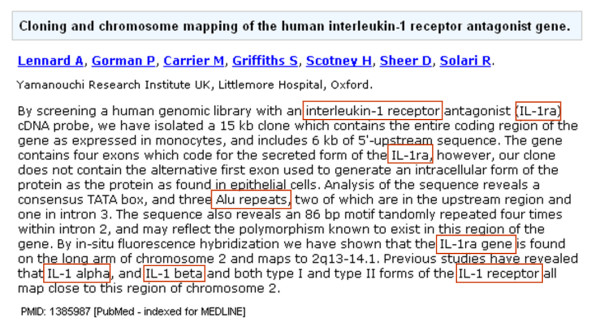
**PubMed document 1385987 annotated with gene/protein mentions**. Title and abstract of a PubMed document annotated with mentions (coloured red) that have been extracted using CBR-Tagger when trained with BioCreative 2 Gene Mention corpus alone.

**Figure 2:**
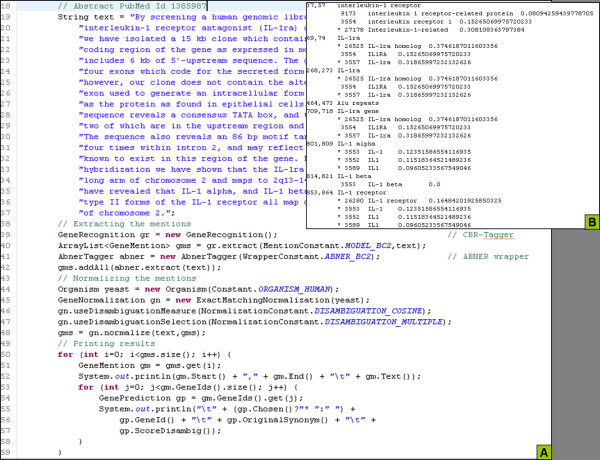
**Code example and output when extracting and normalizing gene/protein mentions**. A: Text extracted from PubMed abstract 1385987 (cf. Figure 1). Extraction was performed with CBR-Tagger and ABNER, both trained with BioCreative 2 Gene Mention corpus alone. Normalization was performed for human using flexible matching and a multiple cosine disambiguation. B: Output presents the text of each extracted mention, including the start and end positions. The gene/protein candidates that were matched to each mention are listed below: the identifier in the Entrez Gene database, the synonym to which the text of the mention was matched, and the disambiguation score. The candidates identified with an asterisk (*) were selected by the system according to the disambiguation strategy. In this example, a multiple disambiguation procedure was used and more than one candidate may be chosen for the same mention.

To demonstrate the functionality of Moara, the abstract of a PubMed document (Figure 1) has been used to extract mentions and normalize them. Figure 2 presents a code example of the extraction and normalization tasks. A free text is provided as the input and the mentions and their respective normalized gene/protein identifiers are returned as an array of the GeneMention objects. In this example we extracted the mentions using both CBR-Tagger and the wrapper of the ABNER tagger which is included in our library (lines 39 to 42). Moara does not extract the title and abstract of the document directly from the Medline repository; reliable, freely available tools can be used for this purpose, such as LingPipe http://alias-i.com/lingpipe/. The GeneMention object encapsulates all the data related to the extracted mentions, the candidates considered during the disambiguation step, and the one (or the ones) that has (have) been chosen as the best candidate(s).

For the normalization function, the array of extracted mentions must be provided, as well as the original text, which is necessary for the disambiguation step. The mentions may be extracted by a tagger, the ones provided at Moara project - ABNER and CBR-Tagger - or any external one. Moara does not restrict the use of any tagger. In the normalization procedure, a matching procedure is carried out and one or more candidates can be chosen, normally the one with highest score (single disambiguation) or the top scored ones according to an automatically defined threshold (multiple disambiguation).

**Figure 3:**
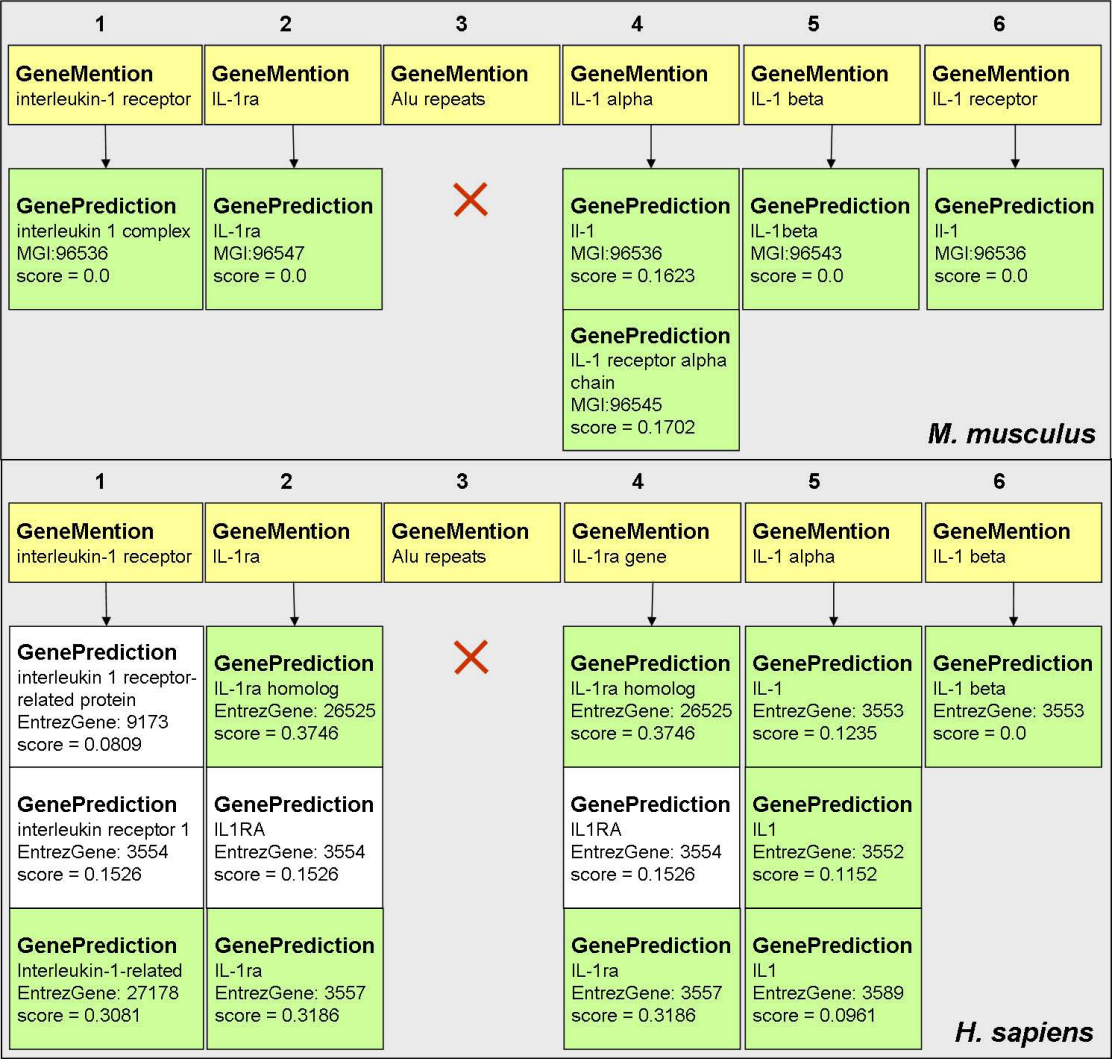
**Results for the code example when normalized to mouse and human**. Gene/protein mentions are coloured yellow; normalization objects are coloured white and green. Mention objects contain the text that was extracted from the document while the normalized objects present the Entrez Gene (human) or MGI (mouse) identifier, the synonym to which the mention text has been matched and the score obtained with the cosine similarity disambiguation strategy. If only one candidate matched the mention, no disambiguation was performed and the score is therefore zero; the higher the score, the better the candidate. The mention "Alu repeats" was not matched to any synonym in the human/mouse dictionaries. Mention "IL-1 beta" was matched to one candidate for both organisms, while other mentions, such as "interleukin -1 receptor", were matched to one candidate for mouse and three candidates for human. For human, mentions 2 and 4 are variations of the same entity and were therefore matched to the same candidates; two of the mentions were chosen by disambiguation analysis. The threshold for multiple disambiguation was automatically calculated for each mention as half the value of the highest score.

Figure 3 illustrates some of the outputs of the example shown in Figure 1, in which one of the mentions, "Alu repeats", returned no normalization; "IL-1 beta" resulted in one candidate; the others were matched to three candidates each due to the multiple disambiguation strategy. A comparison between the mention text and the synonyms to which they have been matched demonstrates the potential of the flexible matching during ML-Normalization. These mentions could have been normalized to another organism by changing the organism's name in line 44 of the code shown in Figure 2. For example, when normalizing the mentions for the mouse, only one candidate is found for most of the mentions and the same mention, "Alu repeats", was not matched to any synonym in the dictionary (Figure 3). However, by normalizing the same mentions to the yeast or fly, no candidates are found.

### Extraction of mentions

Gene/protein recognition is carried out by the CBR-Tagger [16], a tagger based on Cased-based reasoning (CBR) foundations. Case-based reasoning [17] is a machine learning method that consists of learning cases from training documents and retrieving the case most similar to a given problem during the testing step. From this case, the final solution is obtained. One of the advantages of the CBR algorithm is the possibility, by means of checking the features that compose the case-solution, of getting an explanation of why a certain category has been assigned to a given token. In addition, the base of cases can be used as a natural source of knowledge from which to learn extra information about the training dataset, i.e., the number of tokens (or cases) that share a certain value of a feature. Moara provides the possibility of extracting mentions from a text using CBR-Tagger and training it with extra documents. In addition, a wrapper of the ABNER tagger [9] was developed in order to use its mentions without the need to learn the ABNER library.

#### Training the CBR-Tagger

There are five built-in models in the "moara_mention" database; one model trained with the BioCreative 2 Gene Mention task alone and four models trained with the latter in combination with the BioCreative task 1B corpora for the yeast, mouse and fly and the three. This section explains the training strategy of the system and how it can be trained for extra documents.

First, several cases of the classes considered here (gene mention or not) are stored in two bases, one storing known and the other storing unknown cases [18]. The known cases are used by the system to classify tokens that are not new, i.e. tokens that have appeared in the training documents. The attributes used to represent a known case are the token itself, the category of the token (if it is a gene mention or not), and the category of the preceding token (if it is a gene mention or not). Each token represents a single case, and repetition of cases with exactly the same attributes is not allowed. In order to account for repetitions, the frequency of the case is incremented to indicate the number of times that it appears in the training dataset.

The unknown base is used to classify tokens that were not present in the training documents. The unknown cases are built over the same training data used for the known cases. Instead of saving the token itself, a shape of the token is kept in order to allow the system to classify unknown tokens by looking for cases with similar shape. Therefore, as in the known cases, the attributes that have been used to represent the unknown cases are the shape of the token, the category of the token (if it is a gene mention or not), and the category of the preceding token (if it is a gene mention or not). The system saves these attributes for each token in the sentence as an unknown case. As with known cases, no repetition is allowed and instead the frequency of the case is incremented.

The shape of the token is given by its transformation into a set of symbols according to the type of character found: "A" for any upper case letter; "a" for any lower case letter; "1" for any number; "p" for any token in a stopwords list; "g" for a Greek letter; "$" for identifying 3-letter-prefixes and 4-letter-suffixes in a token. For example, "Dorsal" is represented by "Aa", "Bmp4" by "Aa1", "the" by "p", "cGKI(alpha)" by "aAAA(g)", "patterning" by "pat$a" ('$' separates the 3-letter prefix) and "activity" by "a$vity" ('$' separates the 4-letters suffix). The symbol that represents an uppercase letter ("A") can be repeated to take into account the number of letters in an acronym, as shown in the example above. However, the lowercase symbol ("a") is not repeated; suffixes and prefixes are considered instead. These are automatically extracted from each token by considering the last 4 letters and first 3 letters, respectively; they do not come from a predefined list of common suffixes and prefixes.

CBR-Tagger has been trained with the training set of documents made available during the BioCreative 2 Gene Mention task [5] and with additional corpora to improve the extraction of mentions from different organisms. These extra corpora belong to the gene normalization datasets for the BioCreative task 1B [6] corresponding to yeast, mouse and fly gene/protein normalization. These training datasets will be referred to hereafter as CbrBC2, CbrBC2y, CbrBC2m, CbrBC2f and CbrBC2ymf, depending if they are composed by the BioCreative 2 Gene Mention task corpus alone or combined with the BioCreative task 1B corpus for the yeast, mouse, fly or all three, respectively. Two functionalities are available in CBR-Tagger: extraction of the mentions with the built-in models and training a new CBR-Tagger with extra documents.

CBR-Tagger can be trained with extra corpora if the documents are provided in the format used in the BioCreative 2 Gene Mention task, in which the text of the documents and the annotated gene/protein mentions are provided in two distinct files. For example, the sentence below (PubMed 30937) was part of the BioCreative 2 Gene Mention task training corpus identified by P00030937A0119.

*P00030937A0119 SGPT, SGOT, and alkaline phosphatase concentrations were essentially normal in all subjects*.

The mentions that are present in the sentence are listed as follows:

P00030937A0119|0 3|SGPT

P00030937A0119|5 8|SGOT

P00030937A0119|13 31|alkaline phosphatase

The position of the mention in the original text is represented by the position of the first and last characters of the token, with no consideration of the spaces in the original text. Additionally, cases that have been learned for CBR-Tagger beforehand, from the aforementioned five training datasets, can also be considered. CBR-Tagger provides a method for copying cases automatically, without the need to train the tagger for the latter corpora. More than one tagger can be trained, although a short identifier must be provided for use as part of the name of the tables in the database.

The codes below illustrate the training of CBR-Tagger using the data generated by training the tagger with the BioCreative 2 Gene Mention dataset [5], and documents provided in the specified files, in the format discussed above:

. . .

TrainTagger tt = new TrainTagger();

tt.**useDataModel**(MentionConstant.MODEL_BC2);

tt.**readDocuments**("train.in");

tt.**readAnnotations**("annotations.txt");

tt.**train**();

. . .

#### Extraction of mentions with CBR-Tagger

During the testing step, the system searches the known and unknown bases for the case most similar to the problem and a classification decision is given by the class of the case selected as being most similar. The classification procedure works in a similar way to the construction of cases. The text is tokenized and a sliding window is applied in the forward direction and then in the backward direction. In each case, the system keeps track of the category of the preceding token (false at the beginning), gets the shape of the token (according to the symbols described above) and attempts to find a case most similar to it in the base. If more than one case if found, the one with the higher frequency is chosen.

The search procedure is separated into two parts, one for the known cases and another for unknown cases. In this search strategy, priority is given to the known cases. For known cases, the token is saved exactly as it appeared in the training documents, and the classification is more precise than using unknown cases. The system also separates the token into parts in order to classify them individually. Although CBR life cycle [17] allows the re-training of the system with the experience learnt from retrieved cases, the CBR-Tagger does not include this step.

The "moara_mention" database contains five built-in models; one model trained with the BioCreative 2 Gene Mention task alone and in combination with the corpora for the yeast, mouse and fly, and three trained with BioCreative task 1B. Therefore five constants are available according to the set of documents used for training the tagger. Line 40 in Figure 2 shows an example of the function that extracts the mention using the tagger trained with the CbrBC2 dataset. There is no requirement to retrain the system; all these models are included by default in the specified database. The extraction method receives two string arguments: the predefined or user-specific model used to train the tagger and the text from which the mention are to be recognized.

When adding a new organism to Moara, the user does not need to train CBR-Tagger with specific documents; it is possible but not mandatory. We have implemented these specific models for the yeast, mouse and fly because these were the organisms for which annotated corpora are available from BioCreative tasks. The user can always use the CbrBC2 model or any other tagger that is available.

#### Extraction of mentions with ABNER

We have developed a wrapper for the ABNER tagger [9] in order to allow a mix of taggers to be used when extracting mentions, with no need to learn the details of an extra library. ABNER comes with two models based on the corpora of the NLPBA http://www-tsujii.is.s.u-tokyo.ac.jp/GENIA/ERtask/report.html and BioCreative task 1A challenges. We have constructed five more models for ABNER, namely CbrBC2, CbrBC2y, CbrBC2m, CbrBC2f and CbrBC2ymf, by training it with the same datasets that were used for CBR-Tagger. The code below illustrates the use of the ABNER wrapper for a given text:

. . .

AbnerTagger abner = new AbnerTagger(WrapperConstant.ABNER_BC2);

ArrayList<**GeneMention**> gms = abner.**extract**(text);

. . .

### Normalization of mentions

The normalization task is accomplished by ML-Normalization, which consists of a flexible and a machine learning matching approach as well as a disambiguation strategy based on the text under consideration. Organism-specific data previously extracted from the genome databases are also required at this step. More importantly, ML-Normalization uses freely available minimum organism-specific data. This is especially useful if no specifically tailored dictionary is available. The normalization step was trained for the four supported organisms considered here: yeast, mouse, fly and human. For the matching strategy, a flexible and a machine learning based matching were available.

#### Normalizing mentions by flexible matching

Flexible matching is accomplished by exact matching between the mention extracted from the text and the synonyms in the dictionaries. It is flexible because the mention and the synonyms are previously pre-processed by dividing the token according to punctuations, numbers, Greek letters, and BioThesaurus terms, and finally ordering the parts of the token alphabetically. The initial lists of synonyms for the four organisms were available in the two editions of the BioCreative challenge: BioCreative task 1B [6] for yeast, mouse and fly; and BioCreative 2 gene normalization task [7] for humans. The code presented in Figure 2 (line 45 to 48) illustrates the flexible matching normalization for a given text.

For both flexible and machine learning matching, the normalization method receives the array of mentions ("GeneMention" objects) and the original text, which can be used for the disambiguation strategy, as illustrated in Figure 2 (line 48). The output of the normalization procedure is stored in the same array of "GeneMention" objects, and each object can be associated with one or more "GenePrediction" objects that keep track of the candidates that were matched to the respective mention according to the matching strategy under consideration. However, a mention ("GeneMention" object) may have no associated candidates.

#### Using the dictionary of synonyms

We have made available a list of the pre-processed synonyms used in our flexible matching strategy http://moara.dacya.ucm.es/download.html. This allows the option of using our dictionary of synonyms with other matching procedures. However, it should be noted that the same pre-processing procedure must be carried out for the mentions under consideration. We have made available a specific function for this task, which receives the text of the mention and returns a list of variations of the specified text, as shown in the example below:

. . .

Organism yeast = new Organism(Constant.ORGANISM_YEAST);

ExactMatchingNormalization app = new ExactMatchingNormalization(yeast);

String text = "alpha subunit of the rod cGMP-gated channel";

ArrayList<String> variations = app.**getFlexibleMentions**(text);

. . .

The variations of a mention (or synonym) are generated by applying a set of editing procedures to the text, such as breaking the text according to parentheses, numbers and Greek letters, ignoring punctuations and symbols, and filtering tokens such as stopwords and biomedical terms. In order to illustrate the tokenization procedure, the input "YPK1 and YKR2(YPK2) genes" would be separated according to the parenthesis into "YPK1 and YKR2 genes" and "YPK2". The former would be separated into smaller parts, as long as the part is a valid token, i.e., it is not a BioThesaurus term or a stopword. Thus, the "YPK1 and YKR2 genes" would be separated into "YPK1" and "YKR2".

Biomedical terms are filtered in such a way that the number of terms in the BioThesaurus that are ignored from the text is increased according to their frequency in this lexicon. Only those terms with frequencies higher than 10,000 are filtered before the procedure is repeated for terms with frequencies higher than 1,000, 100, 50, 10 or zero (all terms). This procedure generates many variations of the original mention (or synonym).

**Figure 4:**
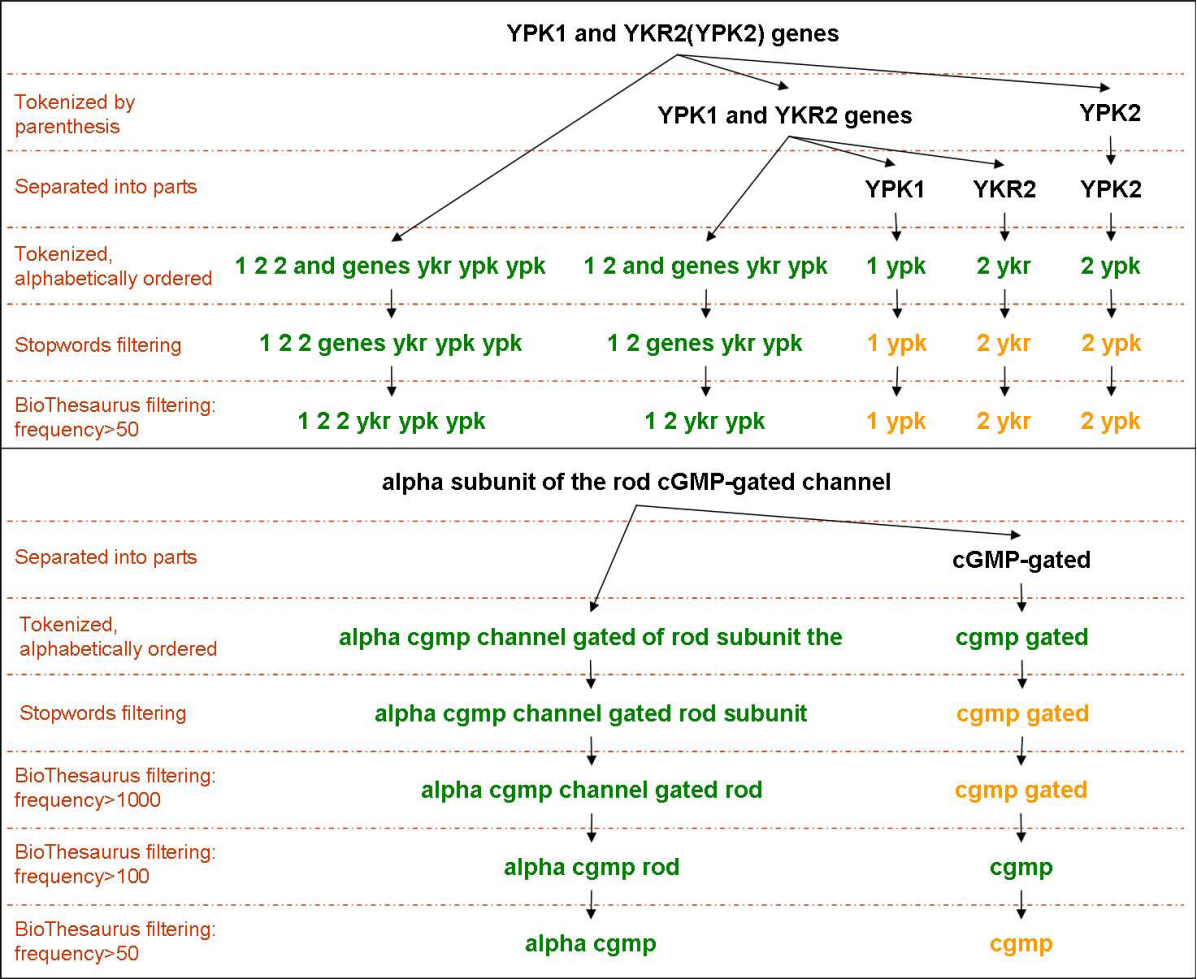
**Editing procedures for the generation of mention and synonym variations**. Two examples of the editing procedures are shown in detail. The non-repeated variations that are returned by the system are presented in green and the repeated variations are shown in orange. Only those procedures that result in a change to the examples are shown. In general, the mentions (or synonyms) are separated according to parenthesis and then into parts that are meaningful on their own. These parts are then tokenized according to numbers, Greek letters and any other symbols (i.e. hyphens), and then the tokens are alphabetically ordered. Gradual filtering is carried out starting with stopwords and followed by the BioThesaurus terms. These are filtered according to their frequency in the lexicon, starting with the more frequent ones (higher than 10,000) to the less frequent ones (at least one).

Figure 4 illustrates the editing procedure for two examples: "YPK1 and YKR2 (YPK2) genes" and "alpha subunit of the rod cGMP-gated channel". The figure has been simplified to include only those steps that generate a new variation of the preceding text in each of the examples. Therefore, the filtering excluded BioThesaurus terms with frequencies higher than 10,000, 10 or zero. The variations shown in green were returned by the system, with no repetition.

Regarding the BioThesaurus, we consider the complete lexicon in our filtering step, i.e., the files identified as "BioMedical terms", "Chemical terms", "Macromolecules" ("enzymes", "single word names" and "general names"), "Common English" and "Single non-word tokens". We perform filtering for the terms identified as "gn" and "pr", as they indicate tokens that refer to genes and proteins.

#### Training of the flexible matching normalization

Moara is trained for using the flexible matching strategy with four organisms: yeast, mouse, fly and human. However, new organisms may be added to the system by providing general available information such as the code of the specified organism in NCBI Taxonomy. For example, in order to train the system for *Bos taurus*, the identifier "9113" must be used. The table "organism" in the "moara" database contains all the organisms present in NCBI Taxonomy. The system will automatically create the necessary tables related to the new organism, including the table that saves information related to the gene/protein synonyms. These tables are easily identified in the database as they are preceded by a nickname such as "yeast" for *Saccharomyces cerevisiae*; in the case of *Bos Taurus*, "cattle" would be an appropriate nickname. Minimum organism-specific information must be provided, for example the "gene_info.gz" and "gene2go.gz" files from Entrez Gene FTP ftp://ftp.ncbi.nih.gov/gene/DATA/, but no gene normalization class needs to be created. An example of training the system for *Bos Taurus *is outlined below:

. . .

Organism cattle = new Organism("9913");

String name = "cattle";

String directory = "normalization";

TrainNormalization tn = new TrainNormalization(cattle);

tn.**train**(name,directory);

. . .

#### Normalizing mentions by machine learning matching

In addition to flexible matching, an approximated machine learning matching is provided for the normalization procedure. The strategy is based on the methodology proposed by Tsuruoka et al. [19], but using the Weka implementation of the Vector Machines (SVM), and Random Forests or Logistic Regression as the machine learning algorithms. In the proposed methodology, the attributes of the training examples are obtained by comparing two synonyms from the dictionary according to predefined features. When the comparison is between two different synonyms for the same gene/protein, it constitutes a positive example for the machine learning algorithm; otherwise, it is a negative example.

The training of the machine learning matching is a three-step procedure in which the data produced in each phase are retained for further use. All the synonyms of its dictionary are represented with the features under consideration, hereafter called "synonym-features": 3-letter-prefix, 3-letters-suffix, a number that is part of the synonym, a Greek letter that is part of the synonym, bigram and trigram and the shape of the synonym, the same features used in the CBR-Tagger. In the second step, pairs of synonyms are chosen on the basis of their similarity, or more precisely, on the percentage of bigrams and trigrams they have in common. This is a time-consuming step and the data obtained are stored for further use. Several experiments have been carried out for different values of the percentage of similarity (0.6, 0.7, 0.8 and 0.9) for both bigram and trigrams. During the third step the system extracts the features that represent the comparison of the synonym-features of the previously selected positive and negative pairs of synonyms, hereafter called "pair-features". These features are indicative of equal prefix, suffix, number and Greek letter, bigram/trigram similarity, string similarity and shape similarity. String similarity is established using the SecondString Java library and experiments have been accomplished for the following string distances [20]: Levenstein, Jaro-Winkler, Smith-Waterman, Monge-Elkan and Soft-TFIDF. These features are used for training the classifiers with one of the available machine learning algorithms: Support Vector Machines, Random Forests or Logistic Regression.

During the testing step, when mentions are presented to be normalized, the system repeats the three-step procedure for each mention: the features of the mentions are extracted (synonym-features); the system selects the candidate synonyms according to a certain percentage of bigram/trigram similarity between the synonyms and the given mention; the features of the selected pairs (pair-features) are extracted to be presented to the machine learning algorithm and to be classified as positive or negative. If a pair of mention-synonyms is classified as positive, the identifier of the respective synonym is set as the gene/protein identifier of the given mention and the normalization task is over. A disambiguation strategy is carried out when more than one pair of mention-synonyms are classified as positive, allowing the best identifier to be chosen from the candidates.

Listed below are the parameters that can be chosen when using machine learning matching for the gene/normalization task:

• Percentage similarity: any value between 0 and 1 (0.9 by default);

• Selection of the pair of mention-synonyms: bigram or trigram similarity, or both (default option);

• Machine learning algorithm: Support Vector Machines (default option), Random Forests or Logistic Regression;

• Set of pair-features: all of them (indicative of equal prefixes, suffixes, numbers and Greek letters, bigram/trigram similarity, string similarity and shape similarity) or just the best of them (bigram/trigram similarity, number and string similarity) (default option).

• String similarity method: Levenstein, Jaro-Winkler, Smith-Waterman (default option), Monge-Elkan or Soft-TFIDF.

The default values shown in the list of parameters above represent the configuration of the system that works reasonably well for the four organisms we have considered (yeast, mouse, fly and human). Therefore, Moara comes with four previously learned models using the default values, one for each of the organisms under consideration. The example below demonstrates how to normalize the previously extracted mention using machine learning matching.

. . .

ArrayList<GeneMention> gms = gr.extract (MentionConstant.MODEL_BC2,text);

MachineLearningNormalization gn = new MachineLearningNormalization(human);

gms = gn.**normalize**(text,gms);

. . .

#### Training of the machine learning matching

Training the machine learning matching is possible for values of parameters outside the built-in models, as well as for new organisms. In the latter case, the procedure to be used is the same as the one presented for flexible matching, with the exception that we must ask the system to generate data for the machine learning matching as well. An example is shown below:

. . .

Organism cattle = new Organism("9913");

String name = "cattle";

String directory = "normalization";

TrainNormalization tn = new TrainNormalization(cattle);

tn.**useMachineLearningNormalization**();

tn.**train**(name,directory);

In order to normalize the mentions using a model based on parameters others than the default ones, the system must first be trained to create the specified model. This procedure can be time-consuming depending on the number of synonyms for the organism under consideration as well as the parameters that have been chosen. The code below demonstrates how to train a model for *Bos taurus *according to the specified parameters:

. . .

Organism cattle = new Organism("9913");

MachineLearningModel mlm = new MachineLearningModel(cattle);

mlm.**setPctSymilarity**(0.6);

mlm.**setFeatures**(NormalizationConstant.NAME_FEATURES_F1);

mlm.**setStringSimilarity**(Constant.DISTANCE_SMITH_WATERMAN);

mlm.**setMachineLearningAlgorithm**(Constant.ML_SVM);

mlm.**setGramSelection**(NormalizationConstant.FEATURE_BIGRAM);

mlm.**train**();

. . .

The "MachineLearningModel" class provides functions for setting any of the parameters discussed above. The system would be ready for normalizing the mentions using the previously trained model. In order that the system uses the model under consideration rather than the default one, the parameters for the "MachineLearningNormalization" class must be explicitly specified, as carried out for the "MachineLearningModel" class. The example below illustrates how to normalize the mention for *Bos taurus *using the previously trained model:

. . .

ArrayList<GeneMention> gms = gr.extractBC2(text);

MachineLearningNormalization gn = new MachineLearningNormalization(human);

gn.**setPctSymilarity**(0.6);

gn.**setFeatures**(NormalizationConstant.NAME_FEATURES_F1);

gn.**setStringSimilarity**(Constant.DISTANCE_SMITH_WATERMAN);

gn.**setMachineLearningAlgorithm**(Constant.ML_SVM);

gn.**setGramSelection**(NormalizationConstant.FEATURE_BIGRAM);

gms = gn.**normalize**(text,gms);

. . .

#### Disambiguation of identifiers

When more than one identifier is obtained for a mention, a disambiguation procedure is used to decide which is more likely to be correct. The selection decision is performed by comparing the similarity between the abstract of the article and a document representative of each of the genes/proteins (gene-document). The gene-document is constructed by compiling information extracted from several databases, such as SGD http://www.yeastgenome.org/ for yeast, MGI http://www.informatics.jax.org/ for mouse, FlyBase http://flybase.org/ for the fly and Entrez Gene http://www.ncbi.nlm.nih.gov/sites/entrez?db=gene for humans. The fields collected for the construction of the gene-documents were symbols, aliases, descriptions, summaries, products, phenotypes, relationships, interactions, Gene Ontology http://www.geneontology.org/ terms related to the gene and their names, definition and synonyms.

Three disambiguation methodologies can be selected. The first considers the cosine similarity [21] between the article and the gene-documents, while the second takes into account the number of common tokens between the two texts. In the first case, the gene-document with the highest cosine similarity is chosen as the correct identifier for the mention. In the second case, the gene-document with highest number of common tokens is chosen as the best solution. The third methodology, based the decisions on both the higher product of the cosine similarity and the number of common tokens, is the default option.

Choosing between single (default option) and multiple disambiguation selection is possible at this step. The single option selects only the best candidate; the multiple selection selects the top scored ones according to a given threshold. The threshold is not a fixed value; it is automatically calculated for each mention and it is given by 50% of the value of the highest score. For example, a mention was matched to four candidates with scores of 0.9, 0.7, 0.5 and 0.4. Using single disambiguation, the only answer is the candidate with best score, 0.9. Using multiple disambiguation, the threshold is automatically calculated as 50% of the highest score, therefore 0.45. The candidates with scores 0.9, 0.7 and 0.5 would be returned by the system as their scores are higher than the threshold. The code of Figure 2 (lines 46-47) shows an example of how to normalize the mention with flexible matching using a disambiguation strategy distinct from the default.

## Results

During development of the system many experiments were carried out in order to decide the final configuration of the system. Experiments concerning gene/protein recognition considered the many corpora that have been used for training CBR-Tagger and the results are presented in Table 1. The best results during the BioCreative 2 Gene Mention task [5] and the results with the ABNER tagger are included in this table. We have trained the ABNER tagger with 15,000 sentences of the training corpus and evaluated over 5,000 sentences of the test dataset. Both the extracted mentions and the evaluation output are available for download at the Moara website http://moara.dacya.ucm.es/download.html. Although the results presented for the gene/protein mention extraction are below the best BioCreative results, this task is considered as a preceding step for gene/protein normalization, and the improvement of this normalization is the main goal of a tagger. Regarding the errors, false negatives in the gene/protein recognition step are not always a problem since the normalization task may be preformed successfully if others (different) mentions of the same gene/protein have been able to be extracted from the text.

**Table 1:** Results for the CBR-Tagger evaluated with the BioCreative 2 GM test set

Training set	Recall	Precision	F-Measure
**CbrBC2**	64.11	76.01	69.56
**CbrBC2y**	42.90	80.98	56.08
**CbrBC2m**	29.14	76.08	42.14
**CbrBC2f**	51.05	73.66	60.30
**CbrBC2ymf**	24.53	77.00	37.21

**Best BioCreative**	85.97	88.48	87.21
**BANNER**	82.78	87.18	84.92
**ABNER**	51.49	86.93	64.68

The BioCreative 2 Gene Mention test set consists of 5,000 sentences. The first five numerical lines represent the results (recall, precision and F-measure) according to the corpus used for training the CBR-Tagger: BioCreative 2 Gene Mention task only (CbrBC2) or combined with the BioCreative 1 task 1B for yeast (CbrBC2y), mouse (CbrBC2m), fly (CbrBC2f) or all three (CbrBC2ymf). The last two lines present the best results of the BioCreative 2 Gene Mention task and BANNER and ABNER results when trained with the latter training corpus.

For the normalization task, we evaluated the best mix of taggers, taking into account ABNER and Banner taggers as well as CBR-Taggers. Experiments were carried out in order to decide the best disambiguation strategy as well as the parameters of the machine learning matching. The results for the normalization task using the configuration of the system that performs best for all the organisms considered here are presented in Table 2. Detailed results for the recognition and normalization tasks as well as an analysis of the mistakes are presented as supplementary material http://moara.dacya.ucm.es/results.html. The best results for yeast and fly were obtained using the BioCreative task 1B [6] and for mouse and human were obtained using GNAT [13]. The GENO [22] system reports an overall F-Measure performance of 86.4 over the BioCreative 2 test set.

**Table 2:** Results for the ML-Normalization evaluated with the test corpora

Organism	Best results (BioCreative and GNAT)			Moara results					
				
				Exact matching			Machine learning matching		
	
	Recall	Precision	F-Measure	Recall	Precision	F-Measure	Recall	Precision	F-Measure
**Yeast**	89.4	95.0	92.1	83.52	95.17	88.97	84.34	81.67	82.99
**Mouse**	91.6	72.6	81.0	77.57	65.83	71.22	79.60	32.90	46.56
**Fly**	80.0	83.1	81.5	69.76	59.12	63.58	69.00	55.22	61.35
**Human**	90.1	81.1	85.4	83.31	55.00	66.26	85.99	29.13	43.52

Best results by organism for the gene/protein normalization task evaluated with the test corpora of the BioCreative 1 task 1B (yeast, mouse and fly) and BioCreative 2 Gene Normalization task (human). These corpora consist of 250 PubMed abstracts each for yeast, mouse and fly, and 262 documents for human. The results were produced using a mix of Abner, Banner and CBR-Tagger (CbrBC2ymf), flexible matching, and single disambiguation by cosine similarity multiplied by the number of common words. The machine learning configuration is the one that performs reasonable well for all the organisms examined here and uses Support Vector Machines as the main algorithm, the F2 set of features (trigram similarity, bigram similarity, number and string similarity), pairs of synonyms selected by 0.9 trigram and bigram similarity and Smith-Waterman for the string similarity feature. The best results for each organism in both competitions are shown.

Although machine learning matching often produces poorer results than exact matching, it is a useful alternative when working with new organisms where the user has no indication of the performance of exact matching. In addition, machine learning produces better recall performance than exact matching, although it is not as precise. In cases where higher recall is needed, machine learning is the best alternative to use.

The results demonstrate that the methodology implemented in Moara is capable of solving gene recognition and normalization tasks in a simple and effective manner. Although CBR-Tagger does not produce the best results when used alone, when combined with other taggers (such as ABNER or BANNER), our experiments (cf. results page) showed that it improves the final results. In the case of normalization approach Moara does not reach the levels of other existing systems. However, as far as we know, no other gene/protein normalization tool is freely available for integrating and for training with new organisms. This is a strong point in Moara since it allows plenty of room for improvements.

Moara utilizes freely available organism-specific data and no tuning was executed for any of the organisms investigated. The possibility of training the system for more organisms makes it a flexible alternative. Therefore, Moara is an asset for those who wish a simple but practical solution to the primary phases of general text mining.

## Conclusions

The Java library presented here represents a good alternative for those scientists working in the text mining field, where gene/protein mention and normalization is required during the process. The performance of Moara is below that achieved in BioCreative competitions, in which the participating systems have made use of specific tailored knowledge for each organism considered, but this is not always available to the scientific community. We have constructed a system that can perform for any organism where minimum organism-specific information is supplied.

We use organism-specific information from different sources and different formats, but no manual curation is performed for the normalization task. Rather than curating the dictionary for each organism, we use those provided by the BioCreative's challenges or by NCBI. Some existing systems [3] use specific tailored information for organisms that produce very good results. However, these dictionaries are not freely available. The free availability and the ease of use of the Java library, as well as the possibility of training the CBR-Tagger and the ML-Normalization with extra documents or organisms, respectively, makes it a necessity for any text mining system.

The inclusion of Moara as an open-source application in Sourceforge is intended to allow the community to change, modify and evolve Moara according to their needs. It is expected that training or using Moara with more specific enriched dictionaries will significantly improve precision and recall. Systems that report an overall gene normalization F-Measure performance of over 80% are not freely available; GENO [22] is not available for download and GNAT [13] is only available through the website. Neither are open-source systems and they are not easily trained, expanded or integrated into another text mining tool such as Moara. As supplementary material http://moara.dacya.ucm.es/suppl_material_bmc.html, a comparative table of the available gene/protein recognition and normalization tools has been built in order to highlight some of the important features of Moara. In view of the importance of integration capabilities, Moara will be fully available in the future in the BioCreative Meta Server [23] and in the U-Compare framework [12], for which the CBR-Tagger is already integrated, and the gene/protein normalization procedure will be extended with other organisms, such as the ones included in Entrez Gene [24] and Uniprot [25]. Some organism-specific databases might also be considered for the most important species, such as HUGO [26] for human.

As this system does not use features that are specific to the gene/protein domain, it might be extended to others domains, although depending on their complexity the CBR-Tagger might be or not appropriate for this task. Some types of entities might require more advanced natural language processing techniques, such as the part-of-speech tag or the use of parsers, while some others are better recognized with the use of specific lexicons, such as the diseases. CBR-Tagger might be more appropriate to those domains which the entities have some similarities to the gene/protein, for example the entities whose names are composed by alphanumeric and symbols or that present some special suffixes or prefixes, in order to exploit the strength of the shape feature.

Moara will be systematically updated to support new functionalities that could potentially help in the analysis of scientific texts. This tool has the potential to help research in this field using and adapting methods that are conceptually simple and powerful for the processing of texts but rather complex in their implementation.

## Availability and requirements

• **Project name**: Moara

• **Project page**: http://moara.dacya.ucm.es/

• **Sourceforge page**: http://sourceforge.net/projects/moara

• **Operating system**: Platform independent

• **Programming language**: Java

• **Other requirements**: MySQL, MySQL JDBC connector, Weka library (optional), SecondString library (optional) and ABNER tagger library (optional).

• **License**: GPL

## Authors' contributions

MLN, JMC and APM conceived and designed the study. MLN designed and developed the software. MLN and APM discussed and implemented the test cases. JMC and APM managed and coordinated the project. All authors participated in writing and revising the final manuscript. All authors have read and approved the final manuscript.
